# Towards Bioengineered Liver Stem Cell Transplantation Studies in a Preclinical Dog Model for Inherited Copper Toxicosis

**DOI:** 10.3390/bioengineering6040088

**Published:** 2019-09-25

**Authors:** Hedwig S. Kruitwagen, Hille Fieten, Louis C. Penning

**Affiliations:** Department of Clinical Sciences of Companion Animals, Faculty of Veterinary Medicine, Utrecht University, 3584CM Utrecht, The Netherlands; H.S.Kruitwagen@uu.nl (H.S.K.); H.Fieten@uu.nl (H.F.)

**Keywords:** copper toxicosis, stem cell transplantation, Wilson Disease, preclinical large animal model

## Abstract

Wilson Disease is a rare autosomal recessive liver disorder in humans. Although its clinical presentation and age of onset are highly variable, hallmarks include signs of liver disease, neurological features and so-called Kayser-Fleischer rings in the eyes of the patient. Hepatic copper accumulation leads to liver disease and eventually to liver cirrhosis. Treatment options include life-long copper chelation therapy and/or decrease in copper intake. Eventually liver transplantations are indicated. Although clinical outcome of liver transplantations is favorable, the lack of suitable donor livers hampers large numbers of transplantations. As an alternative, cell therapies with hepatocytes or liver stem cells are currently under investigation. Stem cell biology in relation to pets is in its infancy. Due to the specific population structure of dogs, canine copper toxicosis is frequently encountered in various dog breeds. Since the histology and clinical presentation resemble Wilson Disease, we combined genetics, gene-editing, and matrices-based stem cell cultures to develop a translational preclinical transplantation model for inherited copper toxicosis in dogs. Here we describe the roadmap followed, starting from the discovery of a causative copper toxicosis mutation in a specific dog breed and culminating in transplantation of genetically-engineered autologous liver stem cells.

## 1. Introduction

The trace element copper is indispensable for various biochemical processes [1]. At the same time, the transition element copper (reduced as Cu^+^ and oxidized as Cu^2+^) is involved in chemical reactions leading to the production of reactive oxygen species. Therefore, its intracellular free concentrations need to be regulated within very narrow boundaries [2]. Regulation occurs at the level of cellular uptake, intracellular binding and distribution, and lastly cellular excretion. Copper is imported into cells mainly via Copper Transporter 1 (Ctr1) [3]. Once copper is inside the cell, copper binding proteins ensure that the free copper levels remain very low. These chaperone proteins include Cytochrome c Oxidase Copper Chaperone (Cox17), Copper Chaperone for Superoxide Dismutase (CCS), and Antioxidant protein1 (ATOX1) [1,2]. Intracellularly copper can be sequestered by glutathione, and metallothionein. Excretion is mediated via P-type ATPases, ATP7A and ATP7B [4]. Transport through the blood stream is mediated via ceruloplasmin. Copper related diseases in humans include Menke’s Disease (copper deficiency disorder), Wilson Disease (copper accumulation), Indian childhood cirrhosis [5], endemic Tyrolean infantile cirrhosis [6], and idiopathic copper toxicosis [7]. The causative mutations for Wilson Disease are in the *ATP7B* gene [8,9]. The ATP7B protein is responsible for excretion of bound intrahepatic copper into the bile. Biliary copper excretion accounts for as much as 95% of the total body copper excretion. There is a wide variation in clinical presentation of Wilson Disease (WD) and a large number of mutations has been reported (well over 500; http://www.wilsondisease.med.ualberta.ca/database.asp) [10,11,12]. Phenotypical variation also occurs within the same genotype, hinting to the effect of modifier genes. In contrast to hepatic copper overload, Menkes Disease (MD) is presented with impaired copper absorption in various organs. Wilson Disease is a rare X-linked copper deficiency disorder caused by mutations in the *ATP7A* gene [13,14,15,16,17]. The limited genotype-phenotype correlation and the rarity of both WD and MD urge for innovative clinical approaches to improve the quality of life of people suffering from Wilson’s or Wilson Disease. What makes dogs (*Canis lupus familiaris*), especially for WD, so well-suited? In order to fully appreciate the potential of these animals, some insights into the canine population structure are necessary. Ever since dogs were domesticated, they have been under severe artificial breeding selection, for instance for behavioral traits and/or specific morphological features [18]. This resulted in isolated genetic populations of dog breeds [19]. The limited genetic variation within breeds, and at the same time a large genetic variation over all breeds, provides a gold-mine for geneticists. Whereas the genetic variation over the various breeds remained intact, the reduced genetic variability within breeds worked as a genetic amplifier and offered researchers a genetic dissection microscope [19]. Together with the selection for a unique trait, such as excessive muscle formation, short limbs or a specific coat color, an increased risk for the development of specific disorders with a simple and/or complex inheritance pattern arose within breeds. Exploiting the downside of inbreeding may therefore be instrumental for the discovery of causative and modifier genes involved in complex diseases and/or rare diseases such as inherited copper toxicosis.

Next to the above mentioned genetic-argument, other research advantages reside within dogs. Dogs are of comparable size of humans and they share similar environmental exposures. Especially the size allows to design and test procedures at a humanized size with highly comparable anatomical arrangements. This is an obvious advantage for preclinical studies, for instance related to liver transplantation. In this respect, the readers might be aware that the first liver transplantations were performed in dogs [20].

In summary both genetic and technical arguments are in favor to utilize dogs as important preclinical models for inherited copper toxicosis. However, there is more to come. Veterinarians have been confronted with sheep and dogs presenting with copper related disorders already for decades [21,22,23,24]. Deleterious levels of hepatic copper are described in several dog breeds including Bedlington terriers, Skye terriers, West-Highland White terriers, Dobermanns, Dalmatians and Labrador retrievers [25,26,27,28,29,30]. Pedigree analysis of most breeds revealed a complex mode of inheritance of copper-mediated hepatitis. Therefore, the phenotypic expression is not dependent on one single genetic factor, but on mutations in more genes and also environmental factors are deemed influential in the phenotypic presentation. As an exception to the rule of thumb that copper toxicosis is a complex genetic disorder, a simple autosomal recessive mode of inheritance is observed in the Bedlington terrier [31].

## 2. A Roadmap towards a Relevant Preclinical Model Animal for Liver Stem Cell Transplantations

**Figure 1:**
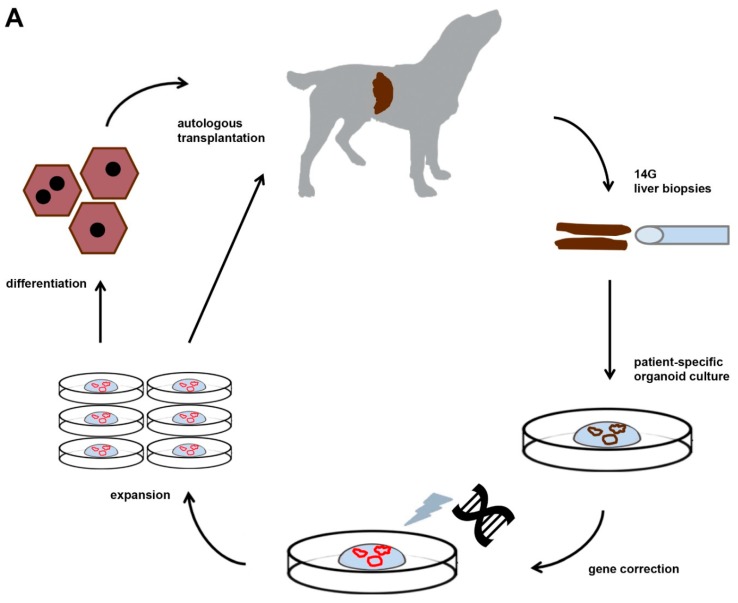
The strategy followed for functional liver recovery after autologous genetically-engineered liver stem cell transplantation in a COMMD1 deficient dog.

Although rodent models for WD have been instrumental to dissect molecularly how mutations in the *ATP7B* gene lead to hepatic copper accumulation, their size does not allow for longitudinal studies (individual animals followed for long time and consecutive measurements). This prompted us to investigate the genetic background of inherited copper toxicosis in several dog breeds. One important feature of a clinical model is the knowledge of the genetic cause and preferentially a simple breeding strategy to acquire sufficient number of experimental animals. In line with this, a similar progression of the disease in time strengthens the validity of the model. Another aspect is the feasibility to obtain sufficient and genetically gene-corrected liver stem cells, preferentially autologous to minimize the risk of rejection of the transplanted cells. Lastly, in view of the clinical application in human medicine, mode of cell transplantation must be similar to the one preferred in human medicine. All these steps will be outlined in more detail below. Figure 1 depicts the strategy followed for functional liver recovery after autologous genetically-engineered liver stem cell transplantation in a COMMD1 deficient dog.

### 2.1. Requirement 1. Copper Accumulation in Bedlington Terriers Is caused by a Deletion of exon-2 in the COMMD1-gene

By means of mapping studies and positional cloning, a 13kB deletion covering exon-2 of the *commd1* gene was discovered as the causative mutation of Bedlington terrier copper toxicosis [31,32].

In the following years, the involvement of this mutation in other copper storage diseases was investigated. It turned out that this mutation was not causative for either Indian Childhood Cirrhosis (ICC), Endemic Tyrolean Infantile Cirrhosis (ETIC), nor Idiopathic copper toxicosis (ICT) [33].

Furthermore, whether or not the *murr1* mutations are somehow involved in WD is a matter of debate [34,35,36]. The intracellular interaction between the ATP7B protein and the COMMD1 protein (previously known as murr1) explains the similarities in WD and Bedlington terrier copper toxicosis [37]. The COMMD1 protein, COpper Metabolism Murr1 domain-containing protein had an unknown function at the time it was discovered. To unravel its function, among others yeast-two hybrid screens were used with COMMD1 as bait. Of interest, a direct COMMD1-ATP7B interaction occurs which was confirmed in cell lines [37]. In WD the interaction between ATP7B and COMMD1 is enhanced and leads to lower ATP7B stability. This interaction partially explains the similar phenotypes of WD in men and copper toxicosis in Bedlington terriers. At present a plethora of functions are related to the COMMD1 protein, including sodium transport via epithelial sodium channel (ENaC), trafficking of cystic fibrosis transmembrane conductance regulator (CFTR), inhibition of Cu/Zn -SOD, NFk-B signalling, Hypoxia Inducing Factor (HIF1) regulation and HIV-replication [37,38,39,40,41]. COMMD1 depletion leads to increased serum Low Density Lipoprotein (LDL) levels, due to mis localization of the LDL-receptor and consequently a reduced uptake of LDL particles [42]. One of the common themes of COMMD1 action seems to be related to protein degradation via ubiquitination, at least regarding NFkB-signalling, ENaC trafficking and HIF1alpha regulation. Results of immunoprecipitated ubiquitinated proteins with associated proteins have not been described, to our knowledge.

Two papers provided direct evidence for a crucial role in cellular copper regulation in in vitro cell cultures [41,43]. By means of siRNAs commd1 was silenced in HEK-293 (human embryonic kidney) cells and in BDE-cells, a canine liver cell line [41,43]. This resulted even in short term cell cultures for increased intracellular copper levels. The fetal lethality of COMMD1 -/- mice is most likely caused by defect in the placenta development, an organ with very high COMMD1 protein expression [43]. Finally, liver specific ATP7B deficient mice had increased hepatic copper levels [44], albeit not as high as the hepatic copper levels in Bedlington copper toxicosis (see Table 1). With respect to hepatic copper accumulation it turned out that COMMD1 protein functions as a chaperone not only for ATP7B, but also for ATP7A [45,46,47].

In summary, the genetic cause of Bedlington terrier copper toxicosis is known, and despite not being in the same gene as for WD, protein–protein interaction of the WD-gene product and the Bedlington-gene product easily explain the similarities in hepatic copper accumulation.

### 2.2. Requirement 2. Longitudinal Studies on COMMD1 Deficient Dogs Highlight Similarities between WD and Canine Copper Toxicosis

All these in vitro and mouse models stimulated us perform longitudinal studies to describe in great detail how copper toxicosis progresses and to which extent this resembles WD. Therefore, an in-house breeding colony of five COMMD1 deficient dogs was followed for over four years. The simple mode of inheritance facilitated us to create a homozygous COMMD1 deficient breed on a Beagle background. 

Biannual liver biopsies were taken for histology, copper measurements, immunohistochemistry, quantitative RT-PCR and Western blotting [48,49,50]. Although these animals are not geno-copies of human WD patients the disease progression at molecular and histological level clearly resembles WD. Variations between WD in men and COMMD1-deficiency mediated copper toxicosis in Bedlington terriers include the amount of copper accumulated (see Table 1) and the absence of neurological features and Kayser–Fleischer rings. Maximum copper accumulation was reached at 12 months of age (adolescence-mid age), which coincided with the first histological signs of hepatitis. At the same time, increased levels of *mt1A* (copper scavenger metallothionein) mRNA were observed. Slightly later, hepatic stellate cells became activated (α-SMA positivity), with increasing reticulin deposition and hepatocytic proliferation in later stages. A further increase over time of histologically confirmed hepatitis and pro-apoptotic caspase-3 activity (first noticed at 18 months) was observed. For further details on the temporal expression of genes involved in copper homeostasis and antioxidant mechanisms like *atox1* (antioxidant 1 copper chaperone), *ccs* (copper chaperone for cytochrome C oxidase), *cox17* (cyclooxygenase 17), *atp7A, atp7B, sod*, *cat*, and *gpx1* readers are referred to previous papers [49,50]. These longitudinal studies clearly established that COMMD1-deficient dogs develop copper-induced chronic liver disease and even cirrhosis in a comparable fashion as do human WD patients. Two minor variations are related to the age of onset which was much more standardized in the dogs and the copper accumulation was more extreme. Together, the important clinical and histological similarities positioned this breeding colony as genetically-defined large animal model to test clinical applicability of new therapeutics developed in rodent models.

### 2.3. Requirement 3. Culture of Sufficient Quantities of COMMD1-Functional Autologous Liver Stem Cells

The liver is one of the few organs acknowledged for its reparative capacity. Replication of differentiated hepatocytes and/or cholangiocytes are responsible for the volume regeneration to compensate for partially removed liver lobes. For this reason, the existence of liver stem cells seemed unnecessary. Some evidence even points to hepatocytes themselves as main sources of liver regeneration [51,52]. To complicate matters even more, several sources of hepatic stem cells are described, presumably depending on the model to induce hepatic damage or their involvement in the hepatocyte renewal during liver organ homeostasis [51,52,53]. Often one of the most obvious histological reactions is a proliferation of a subset of biliary cells, the so-called ductular reaction. Proposed stem cells include, among others, hepatic stellate cells, hepatocytes themselves, or self-renewing pericentral Axin2+ cells that differentiate into polyploid hepatocytes able to replace hepatocyte during homeostatic liver renewal [53,54,55]. Which of these various liver stem cells are involved in the daily wear-and tear of hepatocytes and cholangiocytes and which of these are hampered in their activation in case of severe liver damage is a matter of a fierce debate; the various points of view are summarized [56,57].

The presence and activation of liver progenitor cells was based on experimental mouse models, and ductular reactions (proliferation of intrahepatic bile ducts) were described in various diseases in humans [58,59,60,61,62,63]. Our initial papers described the activation of liver stem cells in canine hepatopathies and compared these patterns to the observation in human hepatopathies [64,65,66,67].

Having established that a ductular reaction occurred in diseased dog livers in a similar fashion as for human liver diseases, we designed experiments to culture canine liver progenitor cells.

Enrichment of liver progenitor cells by means of fluorescent activated cell sorting (FACS) revealed a side population with a gene expression pattern resembling progenitor cells [68].

Interestingly, during the culture of non-purified liver canine liver progenitor cells, on top of non-proliferating hepatocytes colonies of small, progenitor-like cells became visible [69]. Even more interesting these cells differentiated into a hepatocyte-like phenotype (e.g., albumin and MRP2 expression). This stimulated us to dissect pathways involved in their replication in order to obtain sufficient quantities of liver stem cells. Using a siRNA-screen targeting kinases, we established DYRK1A as a novel pathway specific for liver stem cell proliferation [70]. However, interference with harmine, a known inhibitor of DYRK1A, revealed little effect on cell proliferation as it turned out to be crucial for S-phase entry.

A recent development has been the establishment of liver stem cells cultures as 3D cultured organoids. Organoids are an artificially grown mass of (stem) cells resembling an organ’s function. These stem cell based mini-organs are described from various organs including murine and human livers [71,72]. The clinical application of these mini-organs range from diseases modelling, advanced toxicology and pharmacological studies, and application in stem cell transplantations. For liver, dog, rat, and cat liver organoids have been described [73,74,75]. With these 3D in vitro cultures, expandable to almost infinity and at the same genetically stable, the combination of bioengineering and cell-biology becomes a reality. Indeed in 2015, canine liver organoids of COMMD1-deficient dogs were described in which by means of lentiviral transduction a COMMD1 cDNA was inserted, COMMD1 protein expression resumed in these organoids, as did their copper excretion and they survived under high copper culture conditions [73].

In brief, culture of large quantities of autologous liver stem cells with a functional copy of the COMMD1 cDNA was within reach.

### 2.4. Requirement 4. Number and Routing of Genetically-Engineered Autologous Transplanted Donor Cells

Hepatocyte transplantation in dogs was first described in 1996 preceding the clinical application of hepatocyte transplantations in humans [76]. Together with the few studies describing transplantation of healthy liver cells in human Crigler-Najjar syndrome, urea cycle defects and phenylketonuria we could make an estimated guess on the number of cells minimally required for functional liver recovery [77,78,79,80]. A growth stimulus for the transplanted cells was provided by a lobectomy of the recipient liver [80]. In order to avoid increased portal pressure during cell transplantation and to enhance engraftment, the required number of cells was injected via the portal vein on three consecutive days through an implanted port-a-cath system [81]. The portal vein has been the preferred routing in human patients to transplant liver cells in order to correct inborn errors of metabolism. This highlights another advantage of a large animal models over murine models, since portal vein injections in mice are very challenging.

## 3. Transplantation of Autologous COMMD1-Positive Liver Organoids into Copper-Laden Livers of COMMD1 Deficient Dogs

Having fulfilled relevant criteria for a valid preclinical model for stem cell transplantation we sailed out to culture enormous quantities of gene-corrected liver stem cells. In about 12 weeks of culture the number of gene-corrected liver stem cells was obtained. In this study, we are following five individual dogs up to two years after the transplantation, and the dogs’ size permitted longitudinal liver sampling. All dogs tolerated the lobectomy and the subsequent liver cell transplantations.

Apparently the usage of the port-a-cath system did not reveal severe side-effects. At histology no signs of tumor formation caused by the transplanted cell are observed for the post-transplantation time points analyzed thus far.

## 4. Bedlington Terriers with a *commd1* Mutation or Labrador Retrievers with an *ATPB* Mutation, Which Is the Preferred Breed to Study WD?

Biomedical researchers are well aware of the fact that each model has its limitations, for instance it is a simplification or exaggeration of the reality. In other words, the beauty (and relevance) of a model is in the eye of the beholder. It is of utmost importance to make a rational decision on which large animal or which specific breed to be used for various preclinical studies to truly have impact on the quality of life of WD patients. In order to facilitate this decision, for instance if one would like to investigate the effect of novel therapeutic compounds, some aspects of two dog breeds with copper toxicosis are compared with clinical parameters of WD, as summarized in Table 1.

## 5. Future of Novel Preclinical Models, DoGtor Can You Help Me?

The European Commission recently established a reference network for rare liver diseases (ERN-RARE-LIVER). This shows Europe’s perseverance to address the specific issues inherent to rare diseases, including limited research resources, a lack of scientific understanding and importantly a lack of public awareness. In this respect the participation of the Faculty of Veterinary Medicine (Utrecht, the Netherlands) in an EASL-sponsored consortium entitled Regenerative Hepatology is a crucial first step to bridge the scientific and preclinical gap for inherited copper toxicosis. Recently a recovery from acute liver failure without transplantation was reported in a small population (*n* = 5) [82]. Zinc and/or copper chelation contributed to the recovery. Similarly, a high zinc, low copper diet decreased hepatic copper levels in a subset of Labrador retrievers suffering from inherited copper toxicosis [83]. This shows the potential of comparative clinical studies in humans and dogs. However, it must be kept in mind that these animals are both target animal for therapy (pets as patients) and model animals. As for all models they represent a part of the complete picture, one or a few aspects are highlighted. In the case of hepatic stem cell transplantation, this is unlikely to become the main treatment of choice for WD since it will only affect liver function and not likely the neurological aspects.

Often research is of mice and meds, but we should consider for preclinical studies on bioengineered livers the option pets and vets. In other words, a dogmatic shift: bioengineered liver with large animal models will benefit people suffering from rare diseases, such as inherited copper toxicosis.

## Figures and Tables

**Table 1:** Comparison of some parameters for Wilson Disease (WD) patients with potential large animal models such as Bedlington terriers (BT) and Labrador retrievers (LR).

	WD	BT	LR
Gene	*ATP7B*	*COMMD1*	*ATP7B*/*ATP7A*
Mode of inheritance	autosomal recessive	autosomal recessive	complex
Age of onset	variable	adolescence-mid age	adolescence-mid age
Liver pathology	cirrhosis	cirrhosis	cirrhosis
Hepatic Cu (mg/dwl)	<1000	<12,000	<1000
Neurology	impaired	not reported	not reported
Population	rare	rare *	very frequent
Kayser–Fleischer rings	present in 50%	not reported	not reported

mg/dwl means mg copper per kg dry weight liver; * due to negative breeding selection on copper toxicosis, the disease in Bedlington terriers almost disappeared.
